# An Evaluation of Environmental Governance in Urban China Based on a Hesitant Fuzzy Linguistic Analytic Network Process

**DOI:** 10.3390/ijerph15112456

**Published:** 2018-11-04

**Authors:** Xing Gao, Cheng Shi, Keyu Zhai

**Affiliations:** 1 The Bartlett School of Planning, University College London, London WC1E 6BT, UK; xing.gao@ucl.ac.uk; 2 College of Architecture and Urban Planning, Tongji University, Shanghai 200092, China; chengshi@tongji.edu.cn; 3 School of Education, University of Glasgow, Glasgow G12 8QQ, UK; k.zhai.1@research.gla.ac.uk

**Keywords:** environmental governance, HFL-ANP, control indexes, network structure, information

## Abstract

The aim of this study is to evaluate the performance of urban environmental governance by developing hesitant fuzzy linguistic analytic network process (HFL-ANP). The study bridges the gaps in current knowledge in the following ways: the study methodically develops the HFL-ANP method to evaluate and pick the optimal environmental governance strategy from alternatives; theoretically, network structure of evaluation indicators system on environmental governance is constructed, and the objective and subjective information in the evaluation process of environmental governance is combined. In detail, based on the environmental Kuznets curve (EKC) and the pollution haven hypothesis, the paper constructs the evaluation indexes system of environmental governance and takes observation time length into consideration. Then, we choose three urban cases of environmental governance by exploring the existing literature. Furthermore, we develop the HFL-ANP method and apply it to the cases. The study calculates the initial weights of all indexes by using multiplicative consistency of the HFL preference relation, and derives the decision matrix through combining objective information with subjective information of environmental governance. Finally, we come to the following conclusions: ANP network stricture is close to real-world practical problems and provides the basis for HFL-ANP method; HFL-ANP is a very suitable method of assessing environmental governance; and based on the urban cases of environmental governance, Shanghai is the optimal alternative. In addition, this indicator system can only be applied to cities in China, and the administrative hierarchy of policies has not been considered by this method. Thus, future studies should expand this method and indicator network to contain different countries and different administrative hierarchy.

## 1. Introduction 

Since 1978, China has experienced remarkable economic growth and continuous environmental degradation [1,2]. Moreover, environmental pollution has led to economic losses and health issues [3,4]. Especially, environmental pollution has led an estimated 3%–8% annual loss of GDP in China [1], and about 40% of all premature deaths in China in 2010 are attributable to poor air quality [3]. Thus, China’s government has viewed environmental protection as one of the most important tasks, and policy makers have spared no efforts to improve environmental quality and promote sustainable development [2]. For example, Xiamen, Ningbo and Dalian stopped the PX (P-Xylene) projects due to antipollution protests. In order to reduce social conflicts, Shifang in Sichuan Province, and Qidong in Jiangsu Province had to strengthen their pollution regulations. Meanwhile, a fair number of previous studies have shown various kinds of indicators and methods to improve environmental management performance [5,6,7]. However, these studies are based on quantitative or objective data evaluation; for example, many studies regard specific emissions of pollutants as indicators [8,9,10]. For example, Jiangsu Province sets annual emission reduction targets and proposes to promote environmental governance by raising public awareness of environmental protection. However, important qualitative or subjective indicators are ignored or replaced in the evaluation process.

In addition to the above empirical studies, some theories have also focused on environmental governance. The environmental Kuznets curve (EKC) has discussed the relationships between development and environment [11,12], and the pollution haven hypothesis has indicated the enterprises’ behaviours in the process of environmental governance [10,13]. Moreover, most modern econometric analysis has suggested the findings of EKC and the pollution haven hypothesis in China [1,14], yet, few studies design evaluation indicators based on the above theories.

This study will assess environmental governance performance based on the theories of the EKC and the pollution haven hypothesis. In order to include all subjective and objective information in evaluation system, we develop the method of hesitant fuzzy linguistic analytic network process (HFL-ANP) and choose the optimal environmental governance strategy among alternatives of Guangzhou, Shanghai and Beijing cases. The study contributes to the existing studies as follows: constructing the reasonable evaluation indicators system of environmental governance with the network structure; combining the objective and subjective information in the decision-making process of environmental governance; and selecting the optimal environmental governance strategy by using the HFL-ANP method.

In order to assess the performance of environmental governance cases, showing the preference information on the indexes is the most important issue. Fuzzy sets can solve uncertainty and fuzziness of decision information [15]. Meanwhile, when policy makers would like to express their imprecise evaluation by linguistic term sets, the fuzzy linguistic method can improve the reliability and flexibility of decision models [16]. However, in real world, some decision makers are hesitant to choose different linguistics. In this sense, the method of hesitant fuzzy linguistic term set (HFLTS) would be effective to help decision makers expressing their opinions [17]. Therefore, this study takes advantage of HFLTS to represent the decision-making information where we take the hesitant degrees of decision makers into account. 

A lot of methods have been used to evaluate environmental governance, such as Multi-Criteria Decision Analysis (MCDA) [18], and Life Cycle Assessment (LCA) [19], especially, Analytic Network Process (ANP) [20], which is one of the MCDA. The present study employs ANP to assess the effective of environmental governance cases. ANP is a MCDA tool that takes complex relationships among parameters into consideration [20]. Before developing ANP which is a generalization of AHP, the Analytic Hierarchy Process (AHP) is developed, which is a well-known MCDA technology [21]. Though AHP is conceptually easy to operate, its strict hierarchical structure is not conductive to deal with the complexities of many real-world issues [22]. In order to solve this problem, the ANP model is proposed to help establish the network structure for the indexes and calculate the comprehensive weights of all indexes [23,24]. Moreover, one of the important advantages of the ANP is to use pair-wise comparisons to obtain weights and determine priority indicator in comparison to other weighting approaches in which weights are assigned arbitrarily [22]. In addition, the ANP can help convert subjective evaluation of related weights with respective to a set of overall scores and priority ratio scale, such as preference, importance, or likelihood. Therefore, there are emerging studies on the ANP in the following fields: forest management [25], industrial management [26,27], strategic policy planning [28,29], and economics and finance [30].

However, there are also some disadvantages with the ANP method in existing studies. Firstly, ANP inherits theoretical weaknesses of the assumptions of AHP because it stems directly from the AHP. These weakness include the rank reversal problem, and the priorities derivation method [31]. The possible rank reversal of ANP has been considerably criticized, and many methods have been put forward to try to solve this issue, such as aggregation method and keeping the local priorities unchanged, etc. [32,33]. Fortunately, the research questions in our study do not involve rank reversal. Our aim is to select the best among many alternatives. Thus, when the number of alternatives increases or decreases, we can still pick out the best even though the ranking of other alternatives may have changed. Priorities derivation method and rank reversal are closely related to each other because they refer to the preferences aggregation method from pairwise comparison matrices used in the AHP and ANP. Solving a reversal problem and performing a preferences aggregation with the use of a left eigenvector method should, as a result, produce a reverse sequence of elements which are pairwise-compared in a matrix. Moreover, in our study, we take behavioral variables into account, which can overcome the problem of reverse order [34]. The second challenge is a proper reproduction of assessment scale. In the process of evaluation, experts’ suggestion can be inconsistent and imprecise, especially in decision problems which contain many debated alternatives or criteria, and thus, the AHP/ANP may not provide a correct solution due to decision-makers’ imprecise evaluations [35]. In our study, the policy aims of environmental governance are clear and uncontroversial. Furthermore, our model takes both subject information and object information into account. Above these can help us to minimize the negative impact of experts or policy makers’ actions on the results.

In our study, time length plays an important role in results because environmental governance may present different results on different indexes in different observation time lengths. Thus, our cases have the following characteristics: first, we have to establish a network structure to indicate the interdependence relationships between above control indexes and time length, because the evaluation values of control indexes, including the public, government and enterprises, can change under different observation time length; second, the primary weights of all indexes should be changed into the comprehensive due to the effect of time length on the public, government and enterprises in the decision-making process. Definitely, the ANP approach is better to address above characteristics [24]. Therefore, the present study deals with the performance evaluation of environmental governance cases by ANP method. Meanwhile, we develop a HFL-ANP to combine subjective information with objective information. Following this Introduction, Section 2 provides the methodology used. Section 3 describes and discusses our results. Finally, the conclusions are presented in Section 4.

## 2. Methodology

Based on the pollution haven hypothesis and the EKC, this study analyzes the group benefits in environmental governance, and takes observation time length into account. In above sense, we construct the index system to evaluate the effectiveness of environmental governance. Furthermore, three environmental governance strategies are chosen as the alternatives. Besides, based on these indexes, we develop the HFL-ANP method to evaluate environmental governance cases.

### 2.1. Index System on Environmental Governance

#### 2.1.1. Group Benefits in Environmental Governance

In order to establish a reasonable and comprehensive index system, we employ the pollution haven hypothesis and the EKC to establish the index system. The relationships between development and environment have been widely analyzed based on the norm of the EKC [11,36]. The EKC literature indicates an inverse U-shaped relationship between environmental pollution and per capita income [37]. In addition, a number of empirical studies have shown the presence of EKC for environmental pollution [1,38]. Therefore, the EKC pays attention to the contributions of the public who can promote environmental governance. Pollution haven hypothesis suggests that enterprises with intensive pollution tend to migrate to cities where environmental regulation is weaker [1]. Empirical evidence has supported the hypothesis in China [10,13]. The hypothesis involves two stakeholders—enterprises and government. The existing studies have indicated the role of the government in pollution charges and environmental subsidies [14,39]. Therefore, based on the pollution haven hypothesis and the EKC, the study reveals the three stakeholders (Public, Government and Enterprises) in environmental governance, and verifies their standpoints.

The public wants to obtain a good living environment and high quality of life through better environmental governance. They hope that the living energy price is lower [40], and according to the EKC, they are willing to spend their income to deal with environmental pollution. Furthermore, the trust of the public in government is easily established and broken easily as well, and the public satisfaction is fairly significant for promoting environmental governance [41]. The government is closely related to the improvement of urban environment, and is the main driver of environmental governance. For government, the regulatory costs should be decreased significantly, and the penalties for polluters should be increased [42]. Enterprises are another key part in the environmental governance. On the one hand, the main goal of an enterprise is to make profits, whereas protection of the natural environment is not the main concern for enterprises [43]. On the other hand, in order to increase profits, enterprises have to establish an environmental protection image and undertake corresponding social responsibilities of environmental protection [44]. Moreover, enterprises are also willing to develop or introduce new technology or new energy to reduce the cost of environmental pollution [45]. Table 1 summaries the group benefits in the establishment of environmental governance index system.

The above index system of environmental governance is good for the public, government and enterprises. Consequently, they all support the promotion of environmental governance. However, every group obtains different benefits from environmental governance. Thus, in order to balance different benefits, obtaining a suitable weight of the group at the system is worth discussing. Meanwhile, above index system has no specific assessment indexes from the perspective of the groups to evaluate different environmental governance strategies. In addition, the length of observation time cannot be ignored. The difference in observation time has different impact on the groups under governance strategies. For instance, if the mayor in a city focuses on a long observation time, the overall benefits of the government are the most important, whereas when the mayor pays more attention to a short observation time, the weight of enterprises would be the highest [24]. That is, in a short observation time, enterprises can make quick profits at the cost of environmental sacrifice and have sufficient funds to maintain their environmental image, which can rapidly increase the quantity of economic growth. However, over a long observation period, when the quantity requirement is satisfied, the government starts to pay attention to the quality of economic growth. Moreover, in order to maintain social stability and rule, government has to conduct environmental governance in the long term. Thus, the role of government is highlighted in a long observation time. Therefore, in the process of establishing evaluation index system, the study takes the length of observation time into account.

#### 2.1.2. Specific Indexes

Based on above analysis, we construct the specific evaluation indexes for the environmental governance strategies and list the length of observation time in urban pollution governance: 

(1) Public (C_1_)

Living costs caused by living energy price (C_11_). It is one of the indexes that the public focuses on, and can reflect the public’s choice for different living energies. This index was used to evaluate China’s economy and environment, and the conclusion indicate that carbon emissions call for fewer price distortions [46]. The study uses this index to express the living cost caused by environmental change for the public.

Public participation caused by average income (C_12_). This index can reflect the capacity and willingness of public participation in environmental governance. Due to poor educational experiences and low socioeconomic status, low-income groups cannot obtain the chance of the most basic environmental health [47]. Specifically, poor education acquisition enables low-income groups to lack civic awareness of participating in environmental governance. Low socioeconomic status makes them do not have enough capital to deal with health problems caused by environmental degradation. In addition, environmental governance has different effects in high-income countries, compared with low-income countries [48]. In detail, environmental governance encourages the public to participate in sustainable consumption in high-income countries. Overall, the existing studies have indicated the relationships between income and environmental governance. Thus, we use this index to characterize the capacity and willingness of public participation in environmental governance.

Satisfaction degree (C_13_). It may be viewed as the most comprehensive index to reflect the public’s attitudes to the quality of environmental governance. The environmental governance of enterprises through customer satisfaction is examined [49]. The study using satisfaction degree to evaluate the environmental flows in the Yellow River concludes that satisfaction degree is significant for water resources governance [50].

The above three indexes related to the public are considered as subjective evaluation values in HFLTS for they belong to the emotions expressed by the public.

(2) Government (C_2_)

Environmental regulatory costs (C_21_). One of government’s responsibilities is to regulate environmental pollution, and the regulatory costs demonstrate the administration efficiency of the government [24]. Increasing regulatory costs rises the environmental concerns [51]. (Sources: statistical yearbooks of the three cities)

The penalties for polluters (C_22_). Governments tend to strengthen penalties for the worst polluters [52]. The environmental protection department can improve the performance of environmental governance through adjusting the penalties method for polluters. However, excessive penalties will stifle economic development and innovation enthusiasm.

(3) Enterprises (C_3_)

Gross industrial output value (C_31_). The index is one of the important indicators, reflecting the industrial profitability. The study on the factors impacting strategies for carbon mitigation and industrial carbon emissions found that gross industrial output value is a major contributor to industrial carbon emissions [53] (sources: statistical yearbooks of the three cities and China Industrial Statistics Yearbooks)

Enterprises image investment of environmental protection (C_32_). It is one of the typical indicators for showing the intangible profit and promoting the social awareness of environmental protection. Enterprises can benefit from image of environmental protection, and enterprises image of environmental protection is conducive to sustainable development [54] (sources: China Industrial Statistics Yearbooks and City Commerce and Industry Bureau)

Green patent application counts (C_33_). The findings on the impact of CO_2_ emissions on green patent application and R&D investment indicate insight for the generation of green technology patents [55]. Green patent technology application, as a policy instrument, has emerged to contribute to environmental protection [56]. It is an important index to evaluate the ability of enterprises dealing with industrial pollution (source: State Intellectual Property Office database)

(4) Length of observation time (C_4_)

This study refers to China’s policy “National plan for tackling climate change (2014–2020)” [57], and important document of President Xi Jinping “National ecological environment protection” [58], to define the time length.

Short time (C_41_). It shows that the effectiveness of environmental governance is assessed within six years.

Medium time (C_42_). It states that the effectiveness assessment of environmental governance is conducted between 6 years and 21 years.

Long time (C_43_). It refers to assessing the environmental governance more than 21 years.

The pollution haven hypothesis and the EKC indicate that the public, government and enterprises are the important roles in environmental governance, and thus, they are viewed as the control indexes. However, the time length is important for assessing the performance of environmental governance because environmental governance would present different results on different indexes over various observation time lengths. For instance, if the government or enterprises increase the R&D investment to develop green technology, the expected performance of environmental governance will only be evident after a long time, but this improvement cannot be observed in short time because realizing the market value of green patents needs a long time (at least 20 years, including patent licensing, market value transformation and practical application of technology). In addition, in a short time, the weight of the public should be the highest whereas the weights of enterprises and government decrease, because the decision makers pay more attention to the change of the public’s satisfaction degree, income and cost in the short term. In a medium timeframe, the decision makers underline the development of enterprises, which can increase regional economic growth speedily. Meanwhile, the public attitude is still more important to social stability than the regulatory costs of government. Therefore, the weight of enterprises is higher than that of the public while the weight of government is the lowest. In a long time horizon, the policy effectiveness of government gradually emerges, and decision makers concentrate on the performance of environmental governance in an overall situation. Through the reform of regulatory costs and penalties for polluters, the public and enterprises have begun to adapt themselves to such a new policy. Consequently, the weight of government becomes the highest, and the weights of the public is higher than that of enterprises due to China’s “people oriented” thoughts (the people’s democratic dictatorship of China). Overall, the above discussions underline the importance of observation time length in evaluating the performance of environmental governance. Besides, though the time length greatly affects other control indexes, decision makers may not offer assessment information for alternatives directly because of the difficulty in making a judgement.

**Figure 1:**
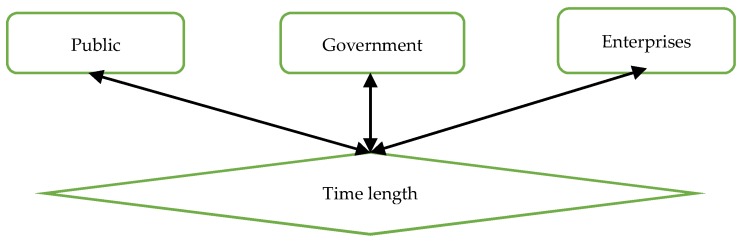
Interdependence relation between control indexes.

Table 2 gives us the evaluation indexes system of environmental governance, and reports the original scale and expected direction of each index in the form of a hierarchical structure. Meanwhile, because the time length (C_4_) is interdependent on other control indexes, all indexes have to be evaluated in the network form to display their interdependence relationship (Figure 1).

#### 2.1.3. Urban Cases of Environmental Governance

After establishing the evaluation indexes system, the study chooses urban environmental governance cases as alternatives from the existing studies, in order to pick the optimal channel for improving the performance of environmental governance. Finally, the study selects three successful cases of urban environmental governance in China, including Guangzhou (A_1_), Shanghai (A_2_) and Beijing (A_3_). They have all made certain achievements in urban environmental governance and extensive literatures have focused on them. 

Table 3 reports the main measures on environmental governance of our alternatives, which are discussed in more detail below: 

Guangzhou. In order to identify the policy preferences of the public, public opinion surveys have been conducted regularly by the municipal government of Guangzhou. More importantly, there is an important trend that the increasing population is focused on pollution issues. In addition, industrial pollution, especially wastewater pollution, has resulted in a noticeable decline in the environmental quality in Guangzhou. Therefore, in 2005, the Guangzhou municipal government conducted a cleanup of the Pear River by a river revitalization proposal. In the process of governance, public participation is required, and penalties for industrial pollution have been strengthened [59,60,61].

Shanghai. It is the most populous and the largest industrial city in China. Since the reform era in 1979, it has faced the formidable task of stopping deterioration of environmental resources, which have placed tremendous stress on industrial production. The municipal authority of Shanghai is in a relatively active position in environmental governance and has organized a policy committee that establishes various kinds of administrative agencies to enforce local environmental policies and regulations. The environmental regulatory costs of Shanghai are very high. According to China Environmental Yearbook 1990–1997, it has spent billions of yuan on environmental governance. In addition, Shanghai conducts environmental impact assessments (EIAs), including governmental policy processes, public participation and enterprises. EIA has made a difference to environmental governance in Shanghai [62,63,64].

Beijing. Beijing has implemented a co-construction and sharing mode of ecological environment governance. In this mode, enterprises need to adjust their industrial structure and upgrade their facilities; the public’s awareness of rights and compensation should be strengthened; and the costs of regulation are decreased by means of taxation. The overall aim of the mode is that a market trading mechanism with government as the core is formed to lighten the burden of government [65,66].

Although the above cities have made great progress in environmental governance, there is no study to compare and analyze the shortcomings and advantages of these cities based on the evaluation index system. Thus, in order to make such a comparison and select the optimal environmental governance model, this study examines Guangzhou, Shanghai and Beijing as alternative approaches.

### 2.2. The HFL-ANP Method to Evaluate Environmental Governance Cases

**Figure 2:**
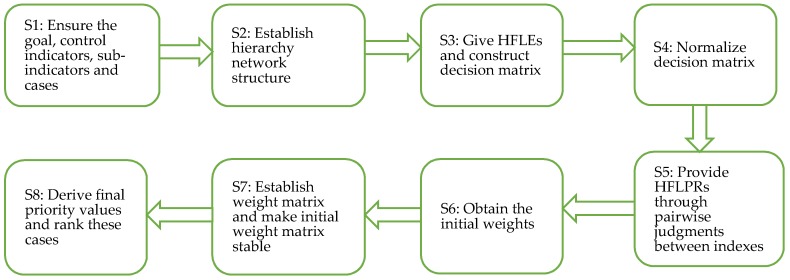
The HFL-ANP process.

The HFL-ANP method is a systematic and practical technique which can be used to select the optimal environmental governance proposal [24]. Also, this method is widely used in the field of medical management and clothing supplier issues [24]. In this study, we follow the steps of the HFL-ANP method to assess the environmental governance cases and choose the best case. The first step is to decompose these environmental governance cases under a hesitant fuzzy linguistic environment, including goals, control indexes, sub-indexes and alternatives. Second, based on the interdependence relations between the public, government, enterprises and time length, the hierarchy structure is established. Third, the study establishes the decision matrix by combining numeric values and HFLEs. Then, in order to construct the preference matrix, we conduct the pairwise comparisons of indexes based on HFLPR (hesitant fuzzy linguistic preference relation). Fourth, the initial weights and weight matrix are obtained. In this process, the weight matrix is limited to make all components in a row be the same. Then, the final priority value and ranking on alternatives can be obtained. Figure 2 depicts the HFL-ANP process which assesses the effectiveness of the environmental governance cases.

The environmental governance evaluation belongs to MCDM issue, including multi-level criteria and alternatives. In terms of ANP, the study decomposes the MCDM problem of environmental governance into network structure. In our study, the environmental governance is divided into four parts: the first is the goal which is used to compare and choose the superior case for environmental governance among three alternatives; the second is control indexes, including the public (C_1_), government (C_2_), enterprises (C_3_) and time length (C_4_); the third is sub-indexes in Table 2; the last is the alternatives, including Guangzhou (A_1_), Shanghai (A_2_) and Beijing (A_3_). As we have discussed, there exist two-way connections between time length and other indexes. In addition, when the decision matrix only includes the judgement on alternatives under the sub-indexes of the public, government and enterprises, the preference information would appear between all control indexes.

Then, we give decision-making information under a hesitant fuzzy linguistic environment. In our study, we consider three categories of information: (1) the pairwise judgement information, being expressed by HFLPR form; (2) the subjective decision-making information, indicating the relationships between some sub-indexes (C_11_, C_22_, C_13_ and C_22_) and alternatives, and being evaluated by HFLEs; (3) the objective decision-making information, reflecting the relationships between alternatives and other sub-indexes (C_21_, C_31_, C_32_, and C_33_), and being calculated by numeric values. Overall, the study aims to strengthen the reliability and diversity of solution by taking the three information types. Unlike the traditional ANP method where the weight vector of sub-indexes is measured by the eigenvector, our study chooses a subjective method to measure the initial weights of indexes.

Though the effectiveness evaluation of environmental governance can be obtained in the existing studies, not all information can be measured by numeric values due to some uncertain and fuzzy components. Using the form of HFLEs can make the decision-making process more accurate and complete [24]. Therefore, copying with the decision matrix should include HFLEs and numeric values. The previous studies have indicated that the HFLEs can be changed into numeric values by the expectation value function and g function [67,68]. Thus, this model is viewed as NVT function:

(1) $$d_{i}=NVT\left(h_{i}\right)=\frac{\sum_{l=1}^{\#h_{i}}g\left(h_{i}\right)}{\#h_{i}}$$

where:

(2) $$g\left(h_{i}\right)=\left\{g\left(s_{\delta_{l}}\right)=\frac{\left(\delta_{l}-4\right)}{2\times 4}+\frac{1}{2}|{\;\delta }_{l}\in \left\{0,1,2,3,\ldots ,8\right\}\right\}\;h_{i}=\left\{s_{\delta_{l}}|\;\delta_{l}\in \left\{0,1,2,3,\ldots ,8\right\},l=1,2,3,\ldots ,\#h_{i}\right\}$$

When $h_{l}=\left\{s_{1},s_{2}\right\}$, the NVT function becomes the following:

(3) $$d_{i}=\frac{\left[g\left(s_{1}\right)+g\left(s_{2}\right)\right]}{2}=\frac{1}{2}\left[\left(\frac{1-4}{2\times 4}+\frac{1}{2}\right)+\left(\frac{2-4}{2\times 4}+\frac{1}{2}\right)\right]=\frac{3}{16}$$

Similarly, when $h_{l}=\left\{s_{1},s_{2},s_{3}\right\}$, the NVT function becomes the following:

(4) $$d_{i}=\frac{\left[g\left(s_{1}\right)+g\left(s_{2}\right)+g\left(s_{3}\right)\right]}{3}=\frac{1}{3}\left[\left(\frac{1-4}{2\times 4}+\frac{1}{2}\right)+\left(\frac{2-4}{2\times 4}+\frac{1}{2}\right)+\left(\frac{3-4}{2\times 4}+\frac{1}{2}\right)\right]=\frac{1}{4}$$

After the HFLEs being transformed into numeric values, the study normalize the decision matrix by the following formulas:

(5) $$For\;positive\;sub-criterion\;x,\;\;\;N_{xy}=\frac{d_{xy}}{\sum_{y=1}^{3}d_{xy}}$$

(6) $$For\;negative\;sub-criterion\;x,\;\;\;N_{xy}=\frac{\frac{1}{d_{xy}}}{\sum_{y=1}^{3}\frac{1}{d_{xy}}}$$

where, x refers to one of the sub-indexes from C, and y is one of the alternatives from A.

Then, we employ a subjective approach to calculate the initial weights of indexes. Due to non-integer numbers in the processing, the discrete linguistic term set should be extended to continuous linguistic term set, that is, $\delta_{l}=\left[0,\;2\tau \right]$ [69]. The multiplicative consistent HFLPR follows:

HFLPR is $B={\left(b_{ij}\right)}_{n\times n}={\left(\left\{b_{ij}^{l}|l=1,2,3,\ldots ,\#b_{ij}\right\}\right)}_{n\times n}\left(i,j=1,2,3,\ldots ,n\right)$, and HFLE is $b_{ij}^{l}=\left\{s_{\delta_{l\left(ij\right)}}|\delta_{i}=\left[0,2\tau \right],\tau =3,l=1,2,3\ldots ,\#b_{ij}\right\}$, where $\#b_{ij}$ refers to the number of linguistic terms of $b_{ij}^{l}$ [69,70]. When the convex feasible region is non-empty, B means the multiplicative consistent HFLPR:

(7) $$\mathrm{Convex}\;\mathrm{feasible}\;\mathrm{region},ℊ=\left\{W={\left(w_{1},w_{2},w_{3},\ldots ,w_{n}\right)}^{T}|\frac{w_{i}}{w_{i}+w_{j}}\in \frac{\delta_{l\left(\mathrm{ij}\right)}}{2\tau },w_{i}>0,i,j=1,2,3,\ldots ,n,\overset{n}{\underset{i=1}{\sum }}w_{i}=1\right\}$$

Where, w refers the weights of indexes. When B is consistent, the study puts forward the following method to measure the subjective weights of indexes based on HFLPR [70]. This method has also been used to measure medical system [24].

(8) $$\frac{w_{i}}{w_{i}+w_{j}}\in \frac{\delta_{l\left(ij\right)}}{2\tau },w_{i}>0,i,j=1,2,3,\ldots ,n$$

For instance:

(9) $$\frac{w_{i}}{w_{i}+w_{j}}\in \delta_{1\left(ij\right)\;}or\;\delta_{2\left(ij\right)\;}or\ldots or\;\delta_{\#b_{ij}\left(ij\right)\;},i,j=1,2,3,\ldots ,n$$

Then, above equation can be changed into:

(10) $$\frac{w_{i}}{w_{j}}=\frac{\delta_{1\left(ij\right)}}{\delta_{1\left(ji\right)}}or\frac{\delta_{2\left(ij\right)}}{\delta_{2\left(ji\right)}}or\ldots or\;\frac{\delta_{\#b_{ij}\left(ij\right)}}{\delta_{\#b_{ij}\left(ji\right)}},i,j=1,2,3,\ldots ,n$$

Because, *w_i_* > 0, *i* = 1,2,3,…,*n*, the study can obtain the following:

(11) $$lnw_{i}-lnw_{j}=ln\frac{\delta_{1\left(ij\right)}}{\delta_{1\left(ji\right)}}\;or\;ln\frac{\delta_{2\left(ij\right)}}{\delta_{2\left(ji\right)}}or\ldots orln\frac{\delta_{\#b_{ij}\left(ij\right)}}{\delta_{\#b_{ij}\left(ji\right)}},i,j=1,2,3,\ldots ,n$$

However, when the deviation increase, above equation is not valid. Then:

(12) $$\varepsilon_{ij}^{l}=lnw_{i}-lnw_{j}-ln\frac{\delta_{l\left(ij\right)}}{\delta_{l\left(ji\right)}},l\in \left\{1,2,3,\ldots ,\#b_{ij}\right\},i,j=1,2,3,\ldots ,n$$

Smaller value of $\varepsilon_{ij}^{l}$ means more accurate the weights of indexes. Thus, the optimization model can be obtained:

(13) $$\min\;Z_{1}=\overset{n}{\underset{i=1}{\sum }}\overset{n}{\underset{j=1}{\sum }}\overset{\#b_{ij}}{\underset{l=1}{\sum }}{\left(\varepsilon_{ij}^{l}\right)}^{2}=\overset{n}{\underset{i=1}{\sum }}\overset{n}{\underset{j=1}{\sum }}\overset{\#b_{ij}}{\underset{l=1}{\sum }}{\left(lnw_{i}-lnw_{j}-\;ln\frac{\delta_{l\left(ij\right)}}{\delta_{l\left(ji\right)}}\right)}^{2}$$

(14) $$s.t.\;\overset{n}{\underset{i=1}{\sum }}w_{i}=1,w_{i}>0,i=1,2,3,\ldots ,n$$

Motivated by the process of heterogeneous incomplete hesitant preference relations [70], the weight can be expressed as follows:

(15) $$w_{i}=\{\begin{array}{c} \frac{exp\left(p_{i}\right)}{\sum_{j=1}^{n-1}exp\left(p_{j}\right)+1},i=1,2,3,\ldots ,n-1\\ \frac{1}{\sum_{j=1}^{n-1}exp\left(p_{j}\right)+1},i=n \end{array}$$

Where, $P=\left(p_{1},p_{2},\ldots ,p_{n-1}\right)=D^{-1}Y$. D and Y are expressed by the following:

(16) $$D=\left[\begin{array}{ccc} \overset{n}{\underset{j=2}{\sum }}l\left(1j\right) & -l\left(12\right)\;\cdots & -l\left(1\left(n-1\right)\right)\\ \vdots & \ddots & \vdots \\ -l\left(\left(n-1\right)1\right) & -l\left(\left(n-1\right)2\right)\cdots & \sum_{j=1,j\neq n-1}^{n}l\left(\left(n-1\right)j\right) \end{array}\right]$$

(17) $$Y=\left[\begin{array}{c} \sum_{j=1}^{n}\sum_{l=1}^{\#b_{1j}}ln\frac{\delta_{l\left(1j\right)}}{\delta_{l\left(j1\right)}}\\ \sum_{j=1}^{n}\sum_{l=1}^{\#b_{2j}}ln\frac{\delta_{l\left(2j\right)}}{\delta_{l\left(j2\right)}}\\ \vdots \\ \sum_{j=1}^{n}\sum_{l=1}^{\#b_{\left(n-1\right)j}}ln\frac{\delta_{l\left(\left(n-1\right)j\right)}}{\delta_{l\left(j\left(n-1\right)\right)}} \end{array}\right]$$

Finally, we construct the supermatrix to calculate the comprehensive weights of control indexes. Due to the interdependences between time length and other indexes, their comprehensive weights are different from their initial weights. The study calculates the pairwise comparison matrices for the public, government and enterprises with respect to different time length, and the pairwise comparison matrices for different time length with respect to the public, government and enterprises. Table 4 gives us an example on the pairwise comparison matrix and the indexes weights for the public, government and enterprises with respect to the short time.

When all indexes weights are consistent, we can obtain the following weight matrix w:

(18) $$w=[\begin{array}{cccccc} w_{1}^{1} & w_{1}^{2} & w_{1}^{3} & w_{1}^{41} & w_{1}^{42} & w_{1}^{43}\\ w_{2}^{1} & w_{2}^{2} & w_{2}^{3} & w_{2}^{41} & w_{2}^{42} & w_{2}^{43}\\ w_{3}^{1} & w_{3}^{2} & w_{3}^{3} & w_{3}^{41} & w_{3}^{42} & w_{3}^{43}\\ w_{41}^{1} & w_{41}^{2} & w_{41}^{3} & w_{41}^{41} & w_{41}^{42} & w_{41}^{43}\\ w_{42}^{1} & w_{42}^{2} & w_{42}^{3} & w_{42}^{41} & w_{42}^{42} & w_{42}^{43}\\ w_{43}^{1} & w_{43}^{3} & w_{43}^{3} & w_{43}^{41} & w_{43}^{42} & w_{43}^{43} \end{array}]$$

Based on above matrix, when indexes C_i_ and C_j_ are independent, $w_{i}^{j}$ is equal to 0. That is, when the public, government and enterprises are independent each other, and when the time length is same, above weight matrix becomes the following:

(19) $$w=[\begin{array}{cccccc} 0 & 0 & 0 & w_{1}^{41} & w_{1}^{42} & w_{1}^{43}\\ 0 & 0 & 0 & w_{2}^{41} & w_{2}^{42} & w_{2}^{43}\\ 0 & 0 & 0 & w_{3}^{41} & w_{3}^{42} & w_{3}^{43}\\ w_{41}^{1} & w_{41}^{2} & w_{41}^{3} & 0 & 0 & 0\\ w_{42}^{1} & w_{42}^{2} & w_{42}^{3} & 0 & 0 & 0\\ w_{43}^{1} & w_{43}^{3} & w_{43}^{3} & 0 & 0 & 0 \end{array}]$$

Replying on above weight matrix, we measure the limit supermatrix (W) to calculate the comprehensive weights of control indexes. Thus, w converges to W as follows:

(20) $$W^{\infty }=\underset{n\to \infty }{lim}w^{2n+1}$$

## 3. Results and Discussion

The aim of HFL-ANP method is to choose the best case from Guangzhou, Shanghai and Beijing.

**Figure 3:**
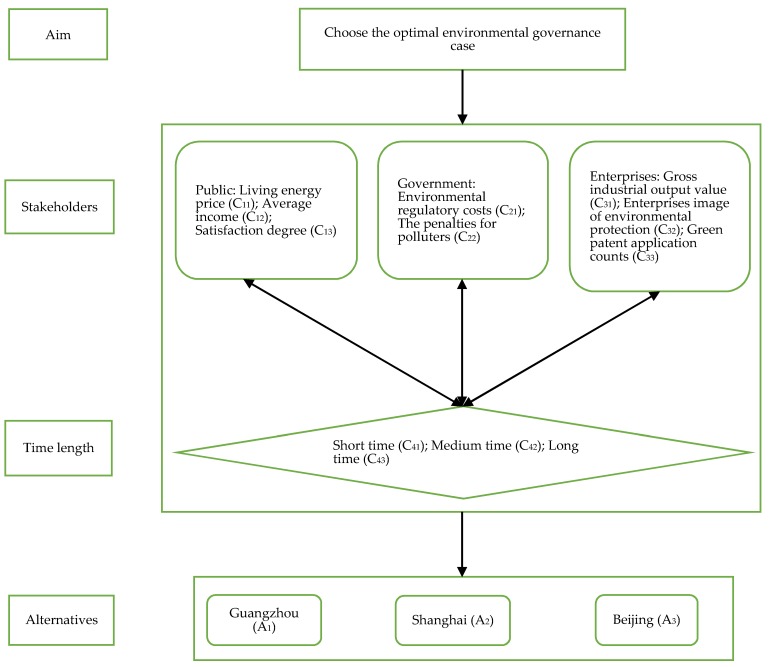
Decomposing the problem of environmental governance issues.

Step 1. Decompose the problem, which is shown in Figure 3.

Step 2. There exists interdependence relationships between the public, government, enterprises and time length. Figure 3 reflects the interdependence structure.

Step 3. Construct the decision matrix of sub-indexes and alternatives, where subjective values and objective values are measured by HFLEs and numeric values respectively. Table 5 reports the decision matrix.

Step 4. The values of HFLEs are changed into numeric values and the decision matrix is normalized by NVT function and normalization algorithm. The following (Table 6) is the normalized decision matrix:

Step 5. On basis of the pairwise judgement information, the preference matrixes of control indexes and sub-indexes are established. The preference matrices for the public, government and enterprise to different time length are reported in R_1_ to R_3_, and the preference matrices for different time length to the public, government and enterprise are given in R_4_ to R_6_.

In addition, the preference matrices for sub-indexes to their control indexes in the public, government and enterprises are indicated from R_7_ to R_9_:
$R_{1}=\begin{array}{ccc} C_{41} & C_{1} & \begin{array}{cc} C_{2} & C_{3} \end{array}\\ C_{1} & \left\{s_{4}\right\} & \begin{array}{cc} \left\{s_{4},s_{3}\right\} & \left\{s_{1}\right\} \end{array}\\ \begin{array}{c} C_{2}\\ C_{3} \end{array} & \begin{array}{c} \left\{s_{4},\;s_{5}\right\}\\ \left\{s_{7}\right\} \end{array} & \begin{array}{cc} \begin{array}{c} \left\{s_{4}\right\}\\ \left\{s_{5}\right\} \end{array} & \begin{array}{c} \left\{s_{3}\right\}\\ \left\{s_{4}\right\} \end{array} \end{array} \end{array}$$R_{2}=\begin{array}{ccc} C_{42} & C_{1} & \begin{array}{cc} C_{2} & C_{3} \end{array}\\ C_{1} & \left\{s_{4}\right\} & \begin{array}{cc} \left\{s_{2}\right\} & \left\{s_{5,}s_{4}\right\} \end{array}\\ \begin{array}{c} C_{2}\\ C_{3} \end{array} & \begin{array}{c} \left\{s_{6}\right\}\\ \left\{s_{3},s_{4}\right\} \end{array} & \begin{array}{cc} \begin{array}{c} \left\{s_{4}\right\}\\ \left\{s_{1}\right\} \end{array} & \begin{array}{c} \left\{s_{7}\right\}\\ \left\{s_{4}\right\} \end{array} \end{array} \end{array}$$R_{3}=\begin{array}{ccc} C_{43} & C_{1} & \begin{array}{cc} C_{2} & C_{3} \end{array}\\ C_{1} & \left\{s_{4}\right\} & \begin{array}{cc} \left\{s_{7}\right\} & \left\{s_{6},s_{5}\right\} \end{array}\\ \begin{array}{c} C_{2}\\ C_{3} \end{array} & \begin{array}{c} \left\{s_{1}\right\}\\ \left\{s_{2},s_{3}\right\} \end{array} & \;\begin{array}{cc} \begin{array}{c} \left\{s_{4}\right\}\\ \left\{s_{5}\right\} \end{array} & \begin{array}{c} \left\{s_{3}\right\}\\ \left\{s_{4}\right\} \end{array} \end{array} \end{array}$$R_{4}=\begin{array}{ccc} C_{1} & C_{41} & \begin{array}{cc} C_{42} & C_{43} \end{array}\\ C_{41} & \left\{s_{4}\right\} & \begin{array}{cc} \left\{s_{3},s_{2}\right\} & \left\{s_{1}\right\} \end{array}\\ \begin{array}{c} C_{42}\\ C_{43} \end{array} & \begin{array}{c} \left\{s_{5},\;s_{6}\right\}\\ \left\{s_{7}\right\} \end{array} & \begin{array}{cc} \begin{array}{c} \left\{s_{4}\right\}\\ \left\{s_{5}\right\} \end{array} & \begin{array}{c} \left\{s_{3}\right\}\\ \left\{s_{4}\right\} \end{array} \end{array} \end{array}$$R_{5}=\begin{array}{ccc} C_{2} & C_{41} & \begin{array}{cc} C_{42} & C_{43} \end{array}\\ C_{41} & \left\{s_{4}\right\} & \begin{array}{cc} \left\{s_{4},s_{3}\right\} & \left\{s_{4}\right\} \end{array}\\ \begin{array}{c} C_{42}\\ C_{43} \end{array} & \begin{array}{c} \left\{s_{4},\;s_{5}\right\}\\ \left\{s_{4}\right\} \end{array} & \begin{array}{cc} \begin{array}{c} \left\{s_{4}\right\}\\ \left\{s_{3}\right\} \end{array} & \begin{array}{c} \left\{s_{5}\right\}\\ \left\{s_{4}\right\} \end{array} \end{array} \end{array}$$R_{6}=\begin{array}{ccc} C_{3} & C_{41} & \begin{array}{cc} C_{42} & C_{43} \end{array}\\ C_{41} & \left\{s_{4}\right\} & \begin{array}{cc} \left\{s_{5}\right\} & \left\{s_{5},s_{4}\right\} \end{array}\\ \begin{array}{c} C_{42}\\ C_{43} \end{array} & \begin{array}{c} \left\{s_{3}\right\}\\ \left\{s_{3},s_{4}\right\} \end{array} & \begin{array}{cc} \begin{array}{c} \left\{s_{4}\right\}\\ \left\{s_{4}\right\} \end{array} & \begin{array}{c} \left\{s_{4}\right\}\\ \left\{s_{4}\right\} \end{array} \end{array} \end{array}$$R_{7}=\begin{array}{ccc} C_{1} & C_{11} & \begin{array}{cc} C_{12} & C_{13} \end{array}\\ C_{11} & \left\{s_{4}\right\} & \begin{array}{cc} \left\{s_{5}\right\} & \left\{s_{4},s_{3}\right\} \end{array}\\ \begin{array}{c} C_{12}\\ C_{13} \end{array} & \begin{array}{c} \left\{s_{3}\right\}\\ \left\{s_{4},s_{5}\right\} \end{array} & \begin{array}{cc} \begin{array}{c} \left\{s_{4}\right\}\\ \left\{s_{6}\right\} \end{array} & \begin{array}{c} \left\{s_{2}\right\}\\ \left\{s_{4}\right\} \end{array} \end{array} \end{array}$$R_{8}=\begin{array}{ccc} C_{2} & C_{21} & C_{22}\\ C_{21} & \left\{s_{4}\right\} & \left\{s_{5}\right\}\\ C_{22} & \left\{s_{3}\right\} & \left\{s_{4}\right\} \end{array}$$R_{9}=\begin{array}{ccc} C_{3} & C_{21} & \begin{array}{cc} C_{22} & C_{23} \end{array}\\ C_{21} & \left\{s_{4}\right\} & \begin{array}{cc} \left\{s_{5},s_{4}\right\} & \left\{s_{5}\right\} \end{array}\\ \begin{array}{c} C_{22}\\ C_{23} \end{array} & \begin{array}{c} \left\{s_{3},s_{4}\right\}\\ \left\{s_{3}\right\} \end{array} & \begin{array}{cc} \begin{array}{c} \left\{s_{4}\right\}\\ \left\{s_{2}\right\} \end{array} & \begin{array}{c} \left\{s_{6}\right\}\\ \left\{s_{4}\right\} \end{array} \end{array} \end{array}$


Step 6. Calculate the initial weights of control indexes through the subjective method. The results (Table 7) follow:

Step 7. Measure the limit supermatrix, and Table 8 reports the final results.

Because there only exist interdependence relationships between stakeholders (the public, government and enterprises) and time length, the initial weights of control indexes are the same as the weights matrix. According to Table 8, the supermatrix does not change when all components in a row is the same and n is equal to 43.

Step 8. The final priority values and the alternatives rank are calculated based on above results. We can obtain the weights of sub-indexes by a subjective approach and the preference relationships reported in R_7_, R_8_ and R_9_. Thus, Table 9 shows the weights results of sub-indexes, the public, government, and enterprises.

The calculation on the weights of control indexes through HFLPR from Step 5 to Step 8 indicates the rank of all indexes: Government > Enterprises > The public, and Long time > Medium time > Short time. Table 8 reports the sequence of sub-indexes’ weights. Regarding the public, Living costs > Public participation > Satisfaction degree; in terms of government, Environmental regulatory costs > The penalties for polluters; for enterprises, Green patent application counts > Gross industrial output value> Enterprises image investment of environmental protection. Finally, we calculate the score of each alternative through sub-indexes weights multiplying their decision values in Table 6. The results are shown in Table 10.

Thus, according to the final scores of these three alternatives in Table 10, the optimal case for environmental governance refers to Shanghai (A_2_), followed by Beijing (A_3_) and Guangzhou (A_1_). In terms of numerical example, the study has assumed that the city pays more attention to the long time observation rather than short time and medium time. Thus, the sequence of control indexes’ weights is government > enterprises > the public from the initial matrix (R_3_), which is same as the results reported by Table 9. Meanwhile, the results in Table 10 also help us understand that the Shanghai (A_2_) performs the best in C_11_ and C_31_ of the control indexes, and behaves pretty well in C_21_, C_22_, C_32_, C_33_, C_13_.

This study postulates that the Shanghai can really improve the performance of environmental governance. The EIA approach of Shanghai not only increases the public participation and public satisfaction of environmental governance, but also stimulates green patent technology innovation and the establishment of environmental image of enterprises. In addition, the policy committee of environmental governance in Shanghai has become an attractive topic. It advocates joint implementation of environmental policies from different governmental departments and participation of different stakeholder [64]. These actions not only increase the penalties for polluters, but also decrease the environmental regulatory costs [63].

The above results also indicate the shortcomings of environmental governance in Beijing and Guangzhou. Regarding Guangzhou, the development of enterprises does not follow a sustainability path, but rather still focuses on the profits. Business growth at the expense of environment can be achieved in the short term, leading to the fact that enterprises are reluctant to spend time on green patent research and development. Though public participation in the process of environmental governance in Guangzhou is required, there are no effective systems and channels to ensure that such behavior occurs. As for Beijing, its main shortcomings are strong political power and high cost of living. Our results indicate that high regulatory costs and low penalties make the environmental protection image of enterprises poor. However, local governments and enterprises do not focus on these issues because enterprises can make profits in the short run and government officials can be promoted in the short term due to the increased GDP [1]. 

In the application process of our method, the weight of observation time length has impact on the final weights of control indexes and all sub-indexes because of the interdependence relationships between time length and control indexes. Underlining different time length may result in different results. In our numerical example, the city attaching more importance to long time length is assumed, and thus, the weight of long time length is the highest, followed by medium time length and short time length. However, if a government is more concerned about the shorter time length, the Shanghai may not the best alternatives. Thus, weights play an important role in selecting the best alternatives. In our study, we test the effect of different time length on results. To change the weights of all observation time length, the study presents different initial preference matrices on time length of control indexes, and the values of other indexes are the same. If the weights of time length follows the order W_42_>W_43_>W_41_, while the preference of matrices is indicated by R_41_, R_51_, R_61:_
$R_{41}=\begin{array}{ccc} C_{1} & C_{41} & \begin{array}{cc} C_{42} & C_{43} \end{array}\\ C_{41} & \left\{s_{4}\right\} & \begin{array}{cc} \left\{s_{3},s_{2}\right\} & \left\{s_{1}\right\} \end{array}\\ \begin{array}{c} C_{42}\\ C_{43} \end{array} & \begin{array}{c} \left\{s_{5},\;s_{6}\right\}\\ \left\{s_{7}\right\} \end{array} & \begin{array}{cc} \begin{array}{c} \left\{s_{4}\right\}\\ \left\{s_{4}\right\} \end{array} & \begin{array}{c} \left\{s_{4}\right\}\\ \left\{s_{4}\right\} \end{array} \end{array} \end{array}$$R_{51}=\begin{array}{ccc} C_{2} & C_{41} & \begin{array}{cc} C_{42} & C_{43} \end{array}\\ C_{41} & \left\{s_{4}\right\} & \begin{array}{cc} \left\{s_{4},s_{3}\right\} & \left\{s_{5}\right\} \end{array}\\ \begin{array}{c} C_{42}\\ C_{43} \end{array} & \begin{array}{c} \left\{s_{4},\;s_{5}\right\}\\ \left\{s_{3}\right\} \end{array} & \begin{array}{cc} \begin{array}{c} \left\{s_{4}\right\}\\ \left\{s_{1},s_{2}\right\} \end{array} & \begin{array}{c} \left\{s_{7},s_{6}\right\}\\ \left\{s_{4}\right\} \end{array} \end{array} \end{array}$$R_{61}=\begin{array}{ccc} C_{3} & C_{41} & \begin{array}{cc} C_{42} & C_{43} \end{array}\\ C_{41} & \left\{s_{4}\right\} & \begin{array}{cc} \left\{s_{4}\right\} & \left\{s_{5},s_{4}\right\} \end{array}\\ \begin{array}{c} C_{42}\\ C_{43} \end{array} & \begin{array}{c} \left\{s_{4}\right\}\\ \left\{s_{3},s_{4}\right\} \end{array} & \begin{array}{cc} \begin{array}{c} \left\{s_{4}\right\}\\ \left\{s_{5}\right\} \end{array} & \begin{array}{c} \left\{s_{3}\right\}\\ \left\{s_{4}\right\} \end{array} \end{array} \end{array}$


If the weights of time length is W_41_ > W_42_ > W_43_, the preference of matrices on different control indexes are R_42_, R_52_, R_62_:
$R_{42}=\begin{array}{ccc} C_{1} & C_{41} & \begin{array}{cc} C_{42} & C_{43} \end{array}\\ C_{41} & \left\{s_{4}\right\} & \begin{array}{cc} \left\{s_{4},s_{3}\right\} & \left\{s_{2}\right\} \end{array}\\ \begin{array}{c} C_{42}\\ C_{43} \end{array} & \begin{array}{c} \left\{s_{4},\;s_{5}\right\}\\ \left\{s_{6}\right\} \end{array} & \begin{array}{cc} \begin{array}{c} \left\{s_{4}\right\}\\ \left\{s_{5}\right\} \end{array} & \begin{array}{c} \left\{s_{3}\right\}\\ \left\{s_{4}\right\} \end{array} \end{array} \end{array}$$R_{51}=\begin{array}{ccc} C_{2} & C_{41} & \begin{array}{cc} C_{42} & C_{43} \end{array}\\ C_{41} & \left\{s_{4}\right\} & \begin{array}{cc} \left\{s_{4},s_{3}\right\} & \left\{s_{6}\right\} \end{array}\\ \begin{array}{c} C_{42}\\ C_{43} \end{array} & \begin{array}{c} \left\{s_{4},\;s_{5}\right\}\\ \left\{s_{2}\right\} \end{array} & \begin{array}{cc} \begin{array}{c} \left\{s_{4}\right\}\\ \left\{s_{2}\right\} \end{array} & \begin{array}{c} \left\{s_{6}\right\}\\ \left\{s_{4}\right\} \end{array} \end{array} \end{array}$$R_{61}=\begin{array}{ccc} C_{3} & C_{41} & \begin{array}{cc} C_{42} & C_{43} \end{array}\\ C_{41} & \left\{s_{4}\right\} & \begin{array}{cc} \left\{s_{6}\right\} & \left\{s_{6},s_{5}\right\} \end{array}\\ \begin{array}{c} C_{42}\\ C_{43} \end{array} & \begin{array}{c} \left\{s_{2}\right\}\\ \left\{s_{2},s_{3}\right\} \end{array} & \begin{array}{cc} \begin{array}{c} \left\{s_{4}\right\}\\ \left\{s_{5}\right\} \end{array} & \begin{array}{c} \left\{s_{3}\right\}\\ \left\{s_{4}\right\} \end{array} \end{array} \end{array}$


Therefore, according to above weights, the different results of environmental governance scores are reported as follows:

According to Table 11, different weights of observation time length have different influence on the sequence of environmental alternatives. In theory, different weights of observation time length are able to change the weights of control indexes due to interrelationship between them. More importantly, the change is illustrated in the process of limiting supermatrix calculation. Consequently, if the government pays attention to a long time period, the optimal city is Shanghai; if the government focuses on medium time, the optimal environmental governance city is Guangzhou; if a short time is chosen by the government, the best option of environmental governance is Beijing.

## 4. Conclusions

The aim of this study is to establish an effective evaluation indexes system for environmental governance and to develop ANP applications in HFLTSs (HFL-ANP). Based on the urban cases of environmental governance from Guangzhou, Shanghai and Beijing, we conclude the following:

Regarding evaluation network construction, the pollution haven hypothesis and the EKC help structure the evaluation indexes system of environmental governance, including the public, enterprise and government. In the process of constructing the evaluation indexes system, the study takes observation time length into account. In addition, the network structure, which connects aim, stakeholders, time length and alternatives of environmental governance, are indicated. The structure is close to real-world practical problems and provides the basis for HFL-ANP method.

In terms of evaluation method, HFL-ANP is a very suitable method to assess environmental governance. The interdependence relationships between different assessment indexes of environmental governance can be shown through a network structure of HFL-ANP method. The comprehensive weights of all indexes, including qualitative and quantitative indicators, can be reported through HFL-ANP method. Moreover, the final results of environmental governance based on the HFL-ANP method can fully reveal complete decision-making information, especially the effect of different observation times.

From the perspectives of final priority values and ranking of alternatives, Shanghai is the optimal alternative. In order to improve the performance of environmental governance, Shanghai conducts EIAs and has policy committees, making it a great success in the C_11_, C_31_, C_21_, C_22_, C_32_, C_33_, and C_13_ areas, however, the result is based on the assumption that the government focuses on a long time horizon of environmental governance. If government officials want to move up in the short term, or businesses want to make profits in the short term, the results will change. In detail, if the government prefers a medium timeframe, Guangzhou is the best alternative, and if short time is underlined, Beijing is the optimal mode.

There are also some limitations on our study. For example, this method and index system are only applicable to Chinese cities because China’s unique political system determines the policy implementation paths and the interdependence between indicators. In addition, this method does not take into account the administrative hierarchy of the government. This point is important because policies at different administrative levels will have an important impact on the performance of environmental governance in China. Future study may combine HFL-ANP method with some optimization methods to solve other environmental governance issues. For instance, the combination between HFL-ANP and simulation method can evaluate the governmental performance of environmental governance at different administrative levels.

## Figures and Tables

**Table 1:** Group benefits in the establishment of environmental governance index system.

Group	Benefits	Support Degree to Environmental Governance
Public (from the EKC)	1. Energy price is reduced [40];	Very supportive
	2. Income is increased to deal with environmental pollution [37];	
	3. The satisfaction of environmental governance is fairly significant [41];	
Government (from the pollution haven hypothesis)	1. The regulatory costs of the government should be significantly decreased [24];	Very supportive
	2. the penalties for polluters should be increased [42];	
Enterprises (from the pollution haven hypothesis)	1. Increasing profits is the main aim, whereas environmental protection is not [43];	Very supportive
	2. The image of social responsibilities of environmental protection should be formed [44];	
	3. New technology or renewable energy should be developed or introduced in order to reduce the cost of pollution [45].	

**Table 2:** Evaluation indexes system of environmental governance.

Control Indexes	Sub-Indexes	Original Scale	Expected Direction
Public (C_1_)	Living costs caused by living energy price (C_11_) [46]	HFLEs	-
	Public participation caused by average income (C_12_) [47,48]	HFLEs	+
	Satisfaction degree (C_13_) [49,50]	HFLEs	+
Government (C_2_)	Environmental regulatory costs (C_21_)[24,51]	N-V	-
	The penalties for polluters (C_22_) [52]	HFLEs	+
Enterprises (C_3_)	Gross industrial output value (C_31_) [53]	N-V	Depends
	Enterprises image investment of environmental protection (C_32_) [54]	N-V	+
	Green patent application counts (C_33_) [55,56]	N-V	+
Time length (C_4_)	Short time (C_41_)	Depends	Depends
	Medium time (C_42_)	Depends	Depends
	Long time (C_43_)	Depends	Depends

Note: HFLEs refers to the form of hesitant fuzzy linguistic, and N-V shows the form of numeric-value scale.

**Table 3:** Alternatives’ measures of environmental governance and relative work.

Cities	Measures	Related Work
Guangzhou	Conduct public opinion surveys on environment pollution	[59,60,61]
	Increase population paying attention to pollution issues	
	Deal with serious industrial pollution hazards	
	Strength penalties for industrial pollution	
Shanghai	Organize a policy committee to copy environmental issues	[62,63,64]
	Offer high environmental regulatory costs	
	Conduct environmental impact assessment (EIA) regulation	
Beijing	Conduct co-construction and sharing mode of ecological environment governance	[65,66]
	Adjust their industrial structure and upgrade industries	
	Strength the public’s awareness of rights and compensation	
	Decrease the costs of regulation by taxation	

**Table 4:** Indexes weights with respect to short time.

C_41_	C_1_	C_2_	C_3_	Indexes Weights
C_1_	$\left(b_{11}^{1},b_{11}^{2},\ldots ,b_{11}^{l}\right)$	$\left(b_{12}^{1},b_{12}^{2},\ldots ,b_{12}^{l}\right)$	$\left(b_{13}^{1},b_{13}^{2},\ldots ,b_{13}^{l}\right)$	$w_{1}^{41}$
C_2_	$\left(b_{21}^{1},b_{21}^{2},\ldots ,b_{21}^{l}\right)$	$\left(b_{22}^{1},b_{22}^{2},\ldots ,b_{22}^{l}\right)$	$\left(b_{23}^{1},b_{23}^{2},\ldots ,b_{23}^{l}\right)$	$w_{2}^{41}$
C_3_	$\left(b_{31}^{1},b_{31}^{2},\ldots ,b_{31}^{l}\right)$	$\left(b_{32}^{1},b_{32}^{2},\ldots ,b_{32}^{l}\right)$	$\left(b_{33}^{1},b_{33}^{2},\ldots ,b_{33}^{l}\right)$	$w_{3}^{41}$

**Table 5:** Initial decision matrix.

	C_21_(Million)	C_22_	C_31_	C_32_	C_33_ (Million)	C_11_	C_12_	C_13_
A_1_	8973	$\left\{s_{2}\right\}$	59%	95%	22	$\left\{s_{2,\;}s_{3}\right\}$	$\left\{s_{2}\right\}$	$\left\{s_{5}\right\}$
A_2_	9210	$\left\{s_{2,\;}s_{3}\right\}$	75%	90%	29	$\left\{s_{2}\right\}$	$\left\{s_{3}\right\}$	$\left\{s_{5}\right\}$
A_3_	9680	$\left\{s_{4}\right\}$	63%	79%	33	$\left\{s_{3,\;}s_{4}\right\}$	$\left\{s_{1}\right\}$	$\left\{s_{6}\right\}$

**Table 6:** Normalized decision matrix.

Indicators	C_21_	C_22_	C_31_	C_32_	C_33_	C_11_	C_12_	C_13_
A_1_	0.37	0.49	0.29	0.38	0.24	0.29	0.25	0.3
A_2_	0.35	0.36	0.41	0.33	0.36	0.51	0.17	0.28
A_3_	0.28	0.15	0.3	0.29	0.4	0.2	0.58	0.42

**Table 7:** Initial weights of control indexes.

Indicators	C_1_	C_2_	C_3_	C_41_	C_42_	C_43_
C_1_	0	0	0	0.15	0.16	0.66
C_2_	0	0	0	0.26	0.73	0.05
C_3_	0	0	0	0.59	0.11	0.29
C_41_	0.07	0.29	0.46	0	0	0
C_42_	0.28	0.48	0.22	0	0	0
C_43_	0.65	0.23	0.32	0	0	0

**Table 8:** Supermatrix convergence.

Indicators	C_1_	C_2_	C_3_	C_41_	C_42_	C_43_
C_1_	0.17	0.17	0.17	0.17	0.17	0.17
C_2_	0.16	0.16	0.16	0.16	0.16	0.16
C_3_	0.16	0.16	0.16	0.16	0.16	0.16
C_41_	0.13	0.13	0.13	0.13	0.13	0.13
C_42_	0.16	0.16	0.16	0.16	0.16	0.16
C_43_	0.22	0.22	0.22	0.22	0.22	0.22

**Table 9:** Comprehensive weights.

Control indexes	Weights	Sub-Indexes	Weights
C_1_	0.15	C_11_	0.45
C_1_	0.15	C_12_	0.36
C_1_	0.15	C_13_	0.19
C_2_	0.21	C_21_	0.67
C_2_	0.21	C_22_	0.34
C_3_	0.19	C_31_	0.28
C_3_	0.19	C_32_	0.25
C_3_	0.19	C_33_	0.46

**Table 10:** Alternatives scores.

Indicators	C_21_	C_22_	C_31_	C_32_	C_33_	C_11_	C_12_	C_13_	Scores
A_1_	0.036	0.031	0.015	0.016	0.028	0.02	0.022	0.012	0.18
A_2_	0.035	0.027	0.029	0.013	0.03	0.025	0.016	0.013	0.188
A_3_	0.029	0.023	0.021	0.011	0.031	0.013	0.038	0.017	0.183

**Table 11:** Final results with respect to different time length weights.

Time Length Sequence	Weight	Scores	Results Sequence
W_43_ > W_42_ > W_41_	W_41_ = 0.12	A_1_ = 0.18	A_2_ > A_3_ > A_1_
	W_42_ = 0.15	A_2_ = 0.188	
	W_43_ = 0.23	A3 = 0.183	
W_42_ > W_43_ > W_41_	W_41_ = 0.12	A_1_ = 0.189	A_1_ > A_2_ > A_3_
	W_42_ = 0.22	A_2_ = 0.178	
	W_43_ = 0.15	A_3_ = 0.177	
W_41_ > W_42_ > W_41_	W_41_ = 0.21	A_1_ = 0.181	A_3_ > A_2_ > A_1_
	W_42_ = 0.16	A_2_ = 0.183	
	W_43_ = 0.13	A_3_ = 0.187	
